# What is the role of plasma cell in the lamina propria of terminal ileum in Good’s syndrome patient?

**DOI:** 10.1515/med-2021-0390

**Published:** 2021-11-08

**Authors:** Yanxia Chen, Weiwei Chen, Yong Wang, Lei Jiang, Jinlin Liu

**Affiliations:** Department of Rheumatology, Zhejiang Provincial People’s Hospital, Hangzhou Medical College, 310014 Hangzhou, China; Department of Anesthesiology, Xinchang Hospital Affiliated to Wenzhou Medical University, 312500 Xinchang, China; Department of Clinical Laboratory, Shangyu People’s Hospital of Shaoxing, 312000, Shangyu, Shaoxing, China; Department of Clinical Laboratory, Zhejiang Provincial People’s Hospital, Hangzhou Medical College, 310014 Hangzhou, China; Department of Clinical Laboratory, Zhejiang Provincial People’s Hospital, Hangzhou Medical College, 158 Shangtang Road, 310014 Hangzhou, China

## To the Editor

We attentively read Chen et al.’s report [1] on the clinicopathologic features of two Good’s syndrome (GS) cases, with diarrhea as a prominent manifestation. Both cases were evaluated by endoscopy, which revealed mucosal edema and fine-granular or nodular appearance changes in the small intestine. Histological examination showed chronic inflammation and villous atrophy. Significantly, author described an interesting finding [1] that the inflammatory cell infiltration in these two GS cases was different. In one case, predominantly CD138^+^ plasma cells with only scattered CD3^+^ T-cell infiltration were revealed in the lamina propria, while in another, it showed predominantly T-cell infiltration without plasma cells in the lamina propria. In contrast, hypoimmunoglobulinemia with low serum levels of IgG, IgA, and IgM and the B cell lineage were all profoundly deficient in the GS patients in this study [1].

Since the original description by Dr Robert Good’s over 60 years ago, most publications of GS were single case reports or small case series. It is characterized by hypogammaglobulinemia, low level or absence of B cells, but the exact cause of hypogammaglobulinemia or decrease of B cells has not, so far, been clarified [2,3]. As we know, B cells are typically considered to be present in the bone marrow throughout the life. Moreover, plasma cells are found in the spleen, lamina propria of the gut, and the bone marrow, with the bone marrow population being highly enriched for long-lived plasma cells [4]. Until now, low level or absence of B cells was mainly reported in the peripheral blood sample, but rarely in other tissue or body fluid in GS patients. To the best our knowledge, only three studies documented that the B cells were significantly suppressed in the bone marrow of GS patients [5,6,7], indicating the defects of B cell precursors in the GS patients. However, whether a marked decrease in the proportion of CD19^+^ B cells in GS is only due to the defect of B cell precursors or includes the defect of mature B cells still need to be investigated.

In the study of Chen et al. [1], HE staining was used to observe the plasma cell in the lamina propria from terminal ileum biopsies, which is shown in Figure 6b (hematoxylin and eosin stain), and CD138 antibody was used to corroborate the plasma cell in Figure 7 (immunohistochemical staining) in one GS patient. Nevertheless, single CD138 antibody staining was not sufficient for the understanding of the role of plasma cells in the lamina propria of terminal ileum in this GS patient. Hence, using CD19, CD20, CD21, CD38, or other B cell lineage markers to investigate the role of B cells in this GS patient is of great interest. Also, the expression of aforementioned B cell lineage markers in the GS patient who lack CD138^+^ plasma cells in the lamina propria is also of interest. Investigation of these B cell lineage markers could help further understand which stage of B cell development is suppressed in GS patient; although the GS is regarded as the second immunodeficiency disease, the exact cause of decrease of B cells has not been clarified [3].

Additionally, as known to all, the intestinal mucosa harbors the largest population of antibody-secreting plasma cells in the human body, which produce daily several grams of IgA [8]. Whether these two GS patients suffered from recurrent diarrhea are associated with the decrease of IgA or other Ig producing intestinal B cell in gut should be investigated in future research [9]:

- Are there abnormalities for intestinal IgA 1,2-, IgM-, and IgG1-4-synthesizing cells in GS patients?
- Do the levels of serum Ig correlate with these Ig-producing cells in the intestine of GS patients?
- Are there abnormalities for J chain synthesis and secretory component in the intestine of GS patients?

Therefore, further immunohistochemistry in this study may provide an important clue for the pathogenesis of GS disease, which could help better understand this rare disease.
